# Large-scale microfluidics providing high-resolution and high-throughput screening of *Caenorhabditis elegans* poly-glutamine aggregation model

**DOI:** 10.1038/ncomms13023

**Published:** 2016-10-11

**Authors:** Sudip Mondal, Evan Hegarty, Chris Martin, Sertan Kutal Gökçe, Navid Ghorashian, Adela Ben-Yakar

**Affiliations:** 1 Department of Mechanical Engineering, The University of Texas at Austin, Austin, Texas 78712, USA; 2 Department of Biomedical Engineering, The University of Texas at Austin, Austin, Texas 78712, USA; 3 Department of Electrical and Computer Engineering, The University of Texas at Austin, Austin, Texas 78712, USA; 4 The Institute for Neuroscience, The University of Texas at Austin, Austin, Texas 78712, USA

## Abstract

Next generation drug screening could benefit greatly from *in vivo* studies, using small animal models such as *Caenorhabditis elegans* for hit identification and lead optimization. Current *in vivo* assays can operate either at low throughput with high resolution or with low resolution at high throughput. To enable both high-throughput and high-resolution imaging of *C. elegans*, we developed an automated microfluidic platform. This platform can image 15 z-stacks of ∼4,000 *C. elegans* from 96 different populations using a large-scale chip with a micron resolution in 16 min. Using this platform, we screened ∼100,000 animals of the poly-glutamine aggregation model on 25 chips. We tested the efficacy of ∼1,000 FDA-approved drugs in improving the aggregation phenotype of the model and identified four confirmed hits. This robust platform now enables high-content screening of various *C. elegans* disease models at the speed and cost of *in vitro* cell-based assays.

## Abstract (web summary)

Large-scale screens on whole animals could facilitate drug discovery, but are technically challenging. Here, Mondal *et al*. develop a microfluidic chip combined with an automated imaging platform that enables high-throughput, high-resolution screening of *Caenorhabditis elegans* disease models.

## Graphical abstract

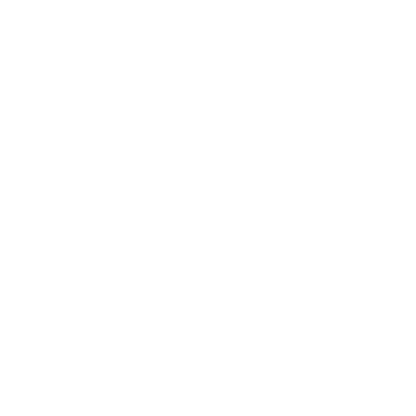

Identification and translation of small-molecule modulators for lead-optimization have been essential tasks in modern drug discovery. The escalating cost during development and clinical trials has been demanding development of new model systems including those based on *in vivo* small animal models. Such *in vivo* systems can recapitulate disease complexity better through drug absorption, distribution, metabolism, excretion, and toxicity^1^,^2^,^3^,^4^.

As one of the best-studied small animal models, *Caenorhabditis elegans* has been used to elucidate molecular pathways and understand disease mechanisms^5^,^6^. *C. elegans*, with roughly 38% homology with human disease genes, has been extensively used to model various disease situations and has the great potential to advance development of effective chemical compounds^7^,^8^. These types of *in vivo* models with highly conserved genomics would be more relevant than *in vitro* cell-based assays, as they can better model disease mechanisms and progression at a whole organism level.

Among the experimental advantages of *C. elegans* are a short life span, well-characterized genetics, a simple neuronal circuit with 302 neurons, a small number of cellular architectures with ∼1,000 cells, and an optically transparent body throughout its development. Continuous advancements in *C. elegans* disease models, such as neurodegenerative^9^,^10^,^11^,^12^,^13^,^14^,^15^, infectious^16^, rare disease^17^, and protein aggregation^15^,^18^, are paving the way for screening large-scale drug libraries on the whole organism level. Current efforts towards the development of cell-specific protein expressions require new high-throughput screening (HTS) platforms operating at higher optical resolutions and speeds than those achievable in currently available technologies.

Current high-speed analysis of *C. elegans* can be performed using low-resolution, flow-based sorting systems such as COPAS Biosort. Such platforms measure the fluorescence signal as integrated across the animal width and monitored along its length with 10 μm resolution as the animal flows through the flow cell^19^,^20^. However, image-based screening methods are necessary to unravel more complex phenotypes where identification of the intensity, shape, and location of features of interest are needed. For imaging, *C. elegans* are conventionally immobilized on agarose pads^21^ or in multi-well plates^22^,^23^ using anaesthetics. Labour-intensive mounting of animals on agar pads results in tedious, low-throughput manual screenings.

Faster imaging, albeit at low resolutions, is possible using plate readers^22^ where cellular phenotypes can be identified rapidly using objectives with low magnifications of 1.6–2.5 × . While high-resolution imaging in plate readers might be possible, however, the random arrangement of the animals imposes slow readout. Such a high-resolution approach requires time-consuming multiple stage motions for finding individual animals in the large area of the wells and for bringing those to the field-of-view (FOV) of the camera and best focal plane. In addition, the collected data will have a large number of empty pixels without useful information.

In recent years, microfluidics have emerged with a promise to overcome these challenges and enable high-throughput studies of *C. elegans* using high-resolution imaging methods^24^,^25^,^26^,^27^,^28^,^29^,^30^,^31^,^32^,^33^,^34^. Integrated with optomechanical systems, microfluidic platforms enable automation by immobilizing the animals in pre-determined locations on the chip. Recent microfluidic studies coupled with automation provided high-resolution imaging of a pair of neurons in a small FOV at speeds of 150–900 animals per hour^35^ and the whole body of *C. elegans* in a larger FOV at speeds of 500 animals per hour using a U-shaped chip configuration^31^. However, these serially operated microfluidic chip configurations can only image animals from a single population. Parallel immobilization chips that can potentially accommodate multiple populations exist. Unfortunately, their complex chip designs prohibited them to expand to larger scales for high-throughput studies^36^,^37^.

Herein, we present the first large-scale microfluidic chip in 96-well format for rapid immobilization, high-resolution imaging, and automated image analysis of multiple populations of *C. elegans* for HTS. The whole chip can immobilize 96 different populations simultaneously within minutes using a chip configuration with 40 parallel traps per population. The novel design of the trap geometry includes reducing dimensions in both height and width to enable rotating the animals while immobilizing them in the desired orientation and at a pre-determined location inside the channels for high-resolution imaging. The densely packed traps allow data efficient image acquisition of all individual animals that are then analysed for aggregation phenotypes using an automated image analysis algorithm. We not only achieved orders of magnitude faster imaging speeds (with 95% filling efficiency, we could image >3,650 animals in 16 min or ∼13,700 animals per hour) compared to the currently available systems, but also overcame the bottleneck problem of studying multiple populations at once. In a pilot study, we used a *C. elegans* model of poly-glutamine aggregation and screened ∼1,000 FDA-approved compounds. The assay provided a Z′-factor of 0.8 and identified four confirmed compounds that significantly reduced aggregation.

## Results

### Design considerations

To achieve high-resolution and high-throughput imaging, we developed a large-scale microfluidic chip for immobilization of a large number of *C. elegans* in a rapid and robust manner. The final design used a single layer microfluidic approach with eight carefully developed unique features: (1) A three-step tapered channel geometry designed to control the aspect ratio for immobilizing day 3 (D3) adult animals in the desired lateral orientation. (2) An un-branched network configuration of parallel traps for increasing speed and density. (3) An optimum spacing between parallel channels for densely packing the chip. (4) Repeating units of eight parallel channels connected to a single exit for simplicity. (5) A 96-well chip format for compatibility with automated liquid handling systems. (6) An on/off cycled pressure driven flow for efficient and rapid trapping of animals. (7) Exit channel widths optimized to accommodate a similar pressure drop for simultaneous immobilization of all animals across the entire chip. (8) Finally, a plug-and-play gasket system for simple operation and compatibility with existing well plate holders. Combined with high-speed image acquisition and image processing algorithms, this chip enabled us to perform HTS of *C. elegans*.

### Poly-glutamine disease model in *C. elegans*

To demonstrate the potential of this imaging platform, we performed a pilot screen using a poly-glutamine (PolyQ) aggregation model in *C. elegans*. We chose this model because of its relevance to Huntington's disease where an abnormal expansion of a CAG repeat in Huntington gene results in an expansion of the PolyQ region that leads to misfolded protein complex, aggregation, and cellular toxicity^38^. Previous studies have quantified the PolyQ length threshold leading to disease symptoms to be 35–40 repeats^15^. A recent cell-based HTS of ∼900,000 small molecules identified 263 proteostasis regulators as primary hits, three of which were demonstrated to enhance protein folding phenotypes at a whole organism level using the PolyQ35 animals with 35 repeat lengths^18^.

The assessment of altering fluorescence features in this *C. elegans* aggregation model however necessitates resolving of small size aggregates, 1–5 μm in diameter, against the background while identifying them among the other nearby features in the whole body of the animal. Previous studies using this model were performed manually and therefore limited to testing of only a few compounds^18^.

### High-resolution imaging platform for HTS of *C. elegans*

Discovery of new molecular pathways or drug-targets using *C. elegans* genetic screening or finding of new drug leads through small molecule screening requires high-resolution imaging and phenotyping of millions of animals from thousands to hundreds of thousands of populations. Such screening involves multiple time-consuming steps, including (1) immobilization of animals, (2) locating individual animals, (3) focusing on the best imaging plane for every animal, (4) acquiring and saving images, and (5) analysing these images for scoring of phenotypes of interest.

To accelerate these steps and achieve high-throughput, we developed a fully automated, high-speed imaging platform for whole body screening of the adult *C. elegans*. This imaging platform includes three parts: (1) a large-scale polydimethylsiloxane (PDMS) microfluidic immobilization chip that simultaneously traps multiple animals from multiple populations in a desired orientation to achieve the best imaging conditions, (2) an automated image acquisition system that acquires high-resolution fluorescence images of whole body of *C. elegans* at high-speed, and (3) an automated image analysis program to score cellular health of each individual animal.

The system also includes an efficient data acquisition and storage process. Because of the large number of images, the amount of data can exceed hundreds of TBs for a large screen, necessitating expensive options for storage and data analysis. Thus, we optimized the chip design and the image acquisition to increase the information density while acquiring images rapidly. First, we designed the chip to include closely placed trapping channels. Second, we designed the image acquisition system with a delicate balance by matching a large area camera with an objective of a moderate magnification and numerical aperture for acquiring the largest FOV possible, while still maintaining micron resolutions.

**Figure 1:**
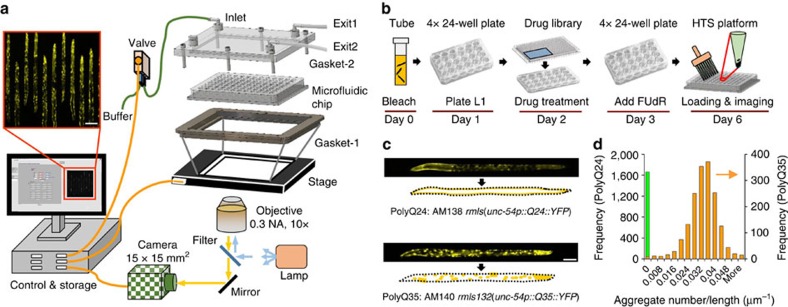
The high-throughput screening (HTS) platform and assay details for *C. elegans* PolyQ aggregation model. (**a**) Schematic of the HTS platform. The 96-well microfluidic chip is operated with a custom designed gasket system to immobilize *C. elegans* inside parallel trapping channels. Scale bar is 200 μm. (**b**) Steps of the new liquid culture (LC) protocol for high-throughput drug treatment and imaging of day 3 adult (D3) animals in the microfluidic chip. (**c**) Images of D3 stage healthy (PolyQ24) and degenerated (PolyQ35) animals immobilized inside microfluidic channels. The animal images are traced manually to show soluble Q24::YFP and punctate Q35::YFP expression present in their body wall muscle cells. Scale bar is 100 μm. (**d**) Frequency distribution of aggregate numbers per length for PolyQ24 (green) and PolyQ35 (orange) animals growing in LC and imaged using the HTS platform indicated on the left and the right axis (orange arrow), respectively (*n*>1,667 animals).

The resulting large-scale microfluidic chip includes 96 conical shaped reservoirs in PDMS, spaced 9 mm apart, with 40 parallel trapping channels per well (Fig. 1a, Supplementary Fig. 1). The chip is clamped within a two-part gasket system to equally pressurize all 96 wells and their associated traps at once. Fluorescence images of the trapped animals are acquired using a 0.3 NA, 10 × magnification objective to resolve the features of interest while allowing optimum imaging density of 10 parallel immobilization channels. Using a large area CCD camera (15 × 15 mm^2^, 2,048 × 2,048 pixels) and 10 × magnification, we can capture a 1.5 mm long portion of the immobilization channels where most trapped animals' whole length are present. The combination of the densely packed trapping channel design and large FOV imaging conditions thus enabled collecting images efficiently with an optimized data density per image at high speed.

After loading the large-scale chip with 96 different populations, the automation software takes over to trap up to 40 animals for each population and performs a quick calibration to estimate the XYZ coordinates of each well from the calibration features on the chip. The software then captures 15 fluorescence z-stack images at 5 μm steps using a piezo stage and saves the images with appropriate file annotations. The image analysis program then loads the z-stacks of the whole FOV, extracts every single animal trapped in the narrow channel, finds the best focal plane for each animal, and finally analyses the images for cellular phenotyping.

We also developed a modified protocol for liquid culturing (LC) of large quantities of *C. elegans* in S medium (Fig. 1b) and treating them with different chemical compounds in parallel for HTS. We characterized the culture conditions using PolyQ aggregation disease model strains PolyQ24, PolyQ35, and PolyQ40 with glutamine repeats of length 24, 35, and 40 residues in body wall muscle cells, respectively^15^,^39^. The animals developed normally in LC and showed similar levels of aggregates as compared to the animals growing on nematode growth medium (NGM) plates (Fig. 1c and Supplementary Fig. 2).

PolyQ24 animals displayed a healthy looking, diffused YFP signal throughout their development until day 5 (D5) adult. In contrast, PolyQ40 animals displayed an aggregated fluorescence distribution starting at an early larval stage. PolyQ35 animals exhibited an age-dependent shift from diffused fluorescence distribution until early adulthood but a more aggregated distribution by D3 adult stage. Since PolyQ40 animals developed slowly and were difficult to grow in large batches, we chose PolyQ35 D3 animals as the disease model for our assay and screened for their phenotype using our microfluidic chip (Fig. 1d). The aggregate parameters, such as the number of aggregates normalized per length of each animal and the standard deviation of the size distribution, were quantified using the automated image analysis program that will be discussed in detail in the following sections. The results showed a clear separation in aggregate distributions between the two populations.

### Large-scale chip design

**Figure 2:**
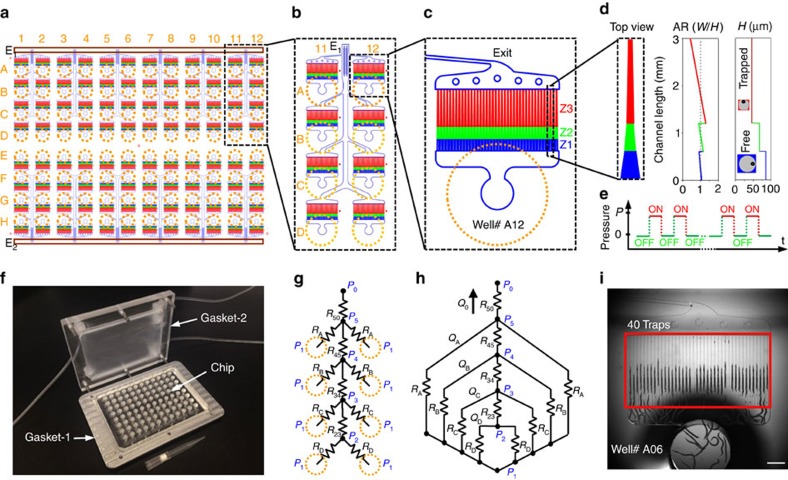
Multi-well microfluidic chip design for *C. elegans* trapping and imaging. (**a**) Schematic of the 96-well chip design with two common exit lines (E1 and E2) along chip edges. (**b**) A repeating unit of 4 × 2 wells connected to the common exit port E1. (**c**) A single well and its immobilization channels with varying aspect ratios (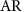
=width/height) in zone 1 (Z1), zone 2 (Z2), and zone 3 (Z3). (**d**) The top view of the trapping channel and its height (*H*) and 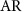 for all three zones as a function of the length. The schematic of the cross-sections of a free and trapped animal is shown at different regions of the trapping channel with the green dots showing the location of their ventral cord position. (**e**) Animals are pushed into the trapping channels using an on/off pressure cycle. (**f**) An image of 96-well chip with the gasket system. (**g**) The map of hydraulic resistances for 40 traps with varying exit channel widths (*R*_A_, *R*_B_, *R*_C_, and *R*_D_) and main exit channel sections (*R*_23_, *R*_34_, *R*_45_, and *R*_50_). (**h**) The equivalent resistance circuit under a common gasket pressure *P*_1_ and resulting flow rates (*Q*_A_, *Q*_B_, *Q*_C_, *Q*_D_, and *Q*_0_). The pressure at the exit port *P*_0_ is assumed to be an atmospheric pressure. (**i**) An image of 40 trapping channels with immobilized animals. Scale bar is 1 mm.

**Figure 3:**
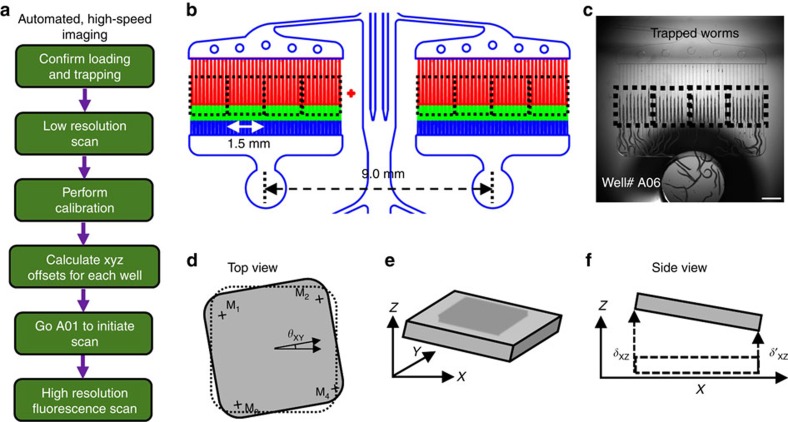
Automation for high-speed image acquisition from multi-well chip. (**a**) Flow chart of the automated imaging. (**b**) Parallel traps are imaged with a large camera to capture a 1.5 × 1.5 mm^2^ portion of the chip with 10 parallel traps and moved in a synchronized manner to capture multiple image stacks at every location. (**c**) An image of well# A06 with all its 40 immobilization channels, showing trapped D3 adult animals and four 1.5 × 1.5 mm^2^ FOVs with 10 parallel traps each (black dotted rectangles). Scale bar is 1 mm. (**d**) Top view of the chip showing four corner markers (M_1_, M_2_, M_3_, and M_4_) and the rotational offset (*θ*_XY_). (**e**) The schematic of the chip overall tilt (*δ*_XY_ and *δ*_XZ_) when mounted on the stage. (**f**) Side view of chip mounted with XZ tilt (*δ*_XZ_).

**Figure 4:**
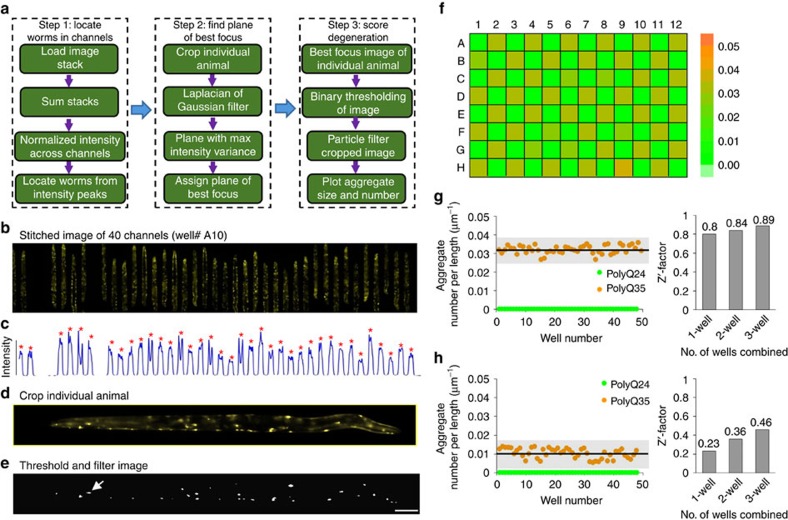
The automated image analysis for estimating the aggregate parameters using high-resolution image stacks and the resulting Z′-factor characterization. (**a**) Workflow of the image analysis algorithms that score degeneration in terms of the aggregate parameters from the image stacks. (**b**) Stitched image of all 40 channels distributed over four FOVs and filled with PolyQ35 animals. (**c**) Normalized intensity profile across the channels. The red dots mark the channels with animals. (**d**) A cropped image of a single channel with an animal. (**e**) Image of the same animal after thresholding and filtering through a particle filter. The arrow indicates one of the aggregates. The scale bar is 100 μm. (**f**) Heat map of median aggregate number per unit length as measured from a whole 96-well chip loaded with a checker box pattern. (**g**) Median aggregate number per unit length of each well quantified using the automated algorithms. The panel on the right shows the Z′-factor for 1, 2, and 3-well average values. (**h**) Median aggregate number per unit length of each well analysed using the lower resolution images (0.13 NA, 4 × objective). The panel on the right shows the Z′-factor for 1, 2, and 3-well average values.

Specific features of the large-scale chip (Fig. 2) included the following elements to fulfil the design considerations: (1) on-chip wells in a standard 96-well format (Fig. 2a), (2) repeating units of eight wells connected to a single exit (Fig. 2b), (3) 40 parallel traps per well (Fig. 2c), (4) an optimum spacing of 150 μm between parallel channels, (5) a three-step tapered channel geometry designed for keeping the channel aspect ratio around the desired ratio of 1 (Fig. 2d), (6) a pressure-driven flow with an on/off loading cycle (Fig. 2e), (7) varying exit channel widths optimized to achieve a similar pressure drop across the entire chip (Fig. 2g,h), and (8) a plug-and-play gasket system with customized outer dimensions to fit in existing well plate holders (Fig. 2f).

A simple but effective way to immobilize the animals relies on the mechanical restriction method using channels that are tapered in their width and organized in a branching network to direct single animals into each trap^36^. Such tapering channel geometry, however, requires 15–30 min to immobilize ∼100 animals, and is too complex to expand to larger scales for high-throughput studies. Moreover, it results in a strongly varying aspect ratio (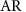
=width/height), rotating the animals to an undesirable random orientation as the aspect ratio reduces below one. To prevent the rotation of animals and keep their natural orientation where the ventral cord is laterally positioned, we decreased the channel height in addition to its width that resulted in three-dimensional (3D) tapered channel geometry. Through several iterations of channel design, we arrived at the optimal dimensions to keep the aspect ratio around one (Fig. 2d).

To immobilize 4,000 animals simultaneously within minutes, we un-branched the network of channels and further developed an on/off pressure driven flow replacing the constant flow-driven loading method used in the previous work. After the initial loading of each population into the wells, the automation software initiates to push the animals into the tapered channels via cycles of on/off pressure. With the optimized geometry, we found that the animals laterally oriented themselves when they were released free during the ‘off' state and could be pushed inside the 3D tapered microchannels in that orientation during the ‘on' state (Fig. 2d, Supplementary Movie 1). After successful immobilization, a constant positive pressure kept animals trapped for high-resolution imaging. A gasket system pressurized all 96 wells simultaneously without any mixing of animals between wells using a single pressure input.

Finally, the simultaneous and efficient loading across such a large-scale chip demanded special design layout and intelligent wiring of the exit channels of individual wells to achieve similar flow rates across the chip. For a configuration with equal exit channel widths, flow rates reduced drastically even across the four tandem channels (Supplementary Fig. 3a). To reduce the effect of well location on flow rates, we equalized the hydraulic resistances (Fig. 2g) by modulating channel widths to compensate for differences in channel lengths. For a given applied pressure *P*_1,_ we iteratively optimized hydraulic resistances by varying channel widths and achieved similar values for the flow rates (*Q*_A_, *Q*_B_, *Q*_C_, and *Q*_D_) using an equivalent circuit (Fig. 2h, Supplementary Fig. 3b–d).

These novel modifications to the tapered channel immobilization method significantly improved the immobilization speed and enabled for the first time to orient the animals in the more desirable lateral orientation, while still keeping them straight. An additional and important benefit of un-branching the network included the ability to densely pack these parallel immobilization channels and thus provided the most efficient image acquisition method. The optimized large-scale microfluidic chip immobilized approximately 3,650 animals simultaneously, filling 95% of the 3,840 trapping channels within 4 min (Fig. 2i, Supplementary Fig. 4).

### Automated image acquisition and storage

For high-speed image acquisition of all 3,840 channels, we developed a LabVIEW-based software synchronizing the motion of translational stages and the image acquisition (Fig. 3a). The software acquires images of all trapped animals automatically across the large-scale chip with an efficient calibration algorithm for achieving the ideal focusing conditions at each location. A flat-top motorized XY stage and a 500 μm range *z* axis piezo stage provide translation rapidly with micrometre and nanometre resolutions in the *x*-*y* and *z* axes, respectively. Before starting imaging of the whole chip, the software performs a quick calibration to identify the best focusing conditions for each location. After imaging specific markers on the chip, the algorithms correct for (1) tilt and rotation of the integrated microfluidic/gasket system with respect to the camera acquisition FOV (Fig. 3b–f) and (2) bending of glass substrate due to the applied pressure during immobilization (Supplementary Fig. 4a–d). After these corrections, the software can acquire a total of 5,760 fluorescence images (96 wells × 4 FOVs/well × 15 z-stack images/FOV) in less than 16 min and store them with appropriate file names for post-image analysis.

### Automated image analysis platform

Manual image analysis of phenotypical changes in large-scale screens is another bottleneck that limits the overall speed of the assay. We developed image processing algorithms to significantly reduce the time required for image analysis (Fig. 4a). A graphical user interface (GUI) was used to simplify the data handling process for large-scale, 3D stacks of images for manual analysis of selected sets of data as necessary for the verification of the automated image analysis program.

For each well, the image processing program first loads all stacks of images for four FOVs and identifies channels with an immobilized animal corresponding to the peaks in the projected intensity profile (Fig. 4b,c). The program then extracts each animal at the best focus plane for image processing (Fig. 4d) and identifies the number of aggregates per unit body length (Fig. 4e). To accommodate day-to-day variation in experimental conditions for each chip, the aggregate features were estimated based on the threshold detected from PolyQ35 animals treated with a vehicle control (0.5% dimethyl sulfoxide, DMSO). Phenotypical scores for each animal are saved automatically in a multi-dimensional array. This program can analyse the aggregation phenotype of all trapped animals in approximately 17 min using nVidia's CUDA with a 3.5 GHz quad core CPU and 6 GB GPU.

### Assay development and Z′-factor characterization

To validate the efficacy of the assay we determined the screening coefficient (Z′-factor). This characterization took into account the entire assay, including the automated imaging program, the optical imaging conditions, the automated image analysis algorithms, and the phenotype scoring.

An example of phenotypical scores from screening of PolyQ24 and PolyQ35 animals grown in LC and imaged on our platform is shown in Fig. 4f,g. Aggregate numbers per unit length of the PolyQ24 and PolyQ35 animals were 0.000±0.000 μm^−1^ and 0.032±0.002 μm^−1^ (median±standard deviation), respectively. This model provided a high screening window coefficient (Z′-factor) of 0.8 for a single well (Fig. 4g), indicating that we achieved the desired consistency and reproducibility for HTS using only one well per compound.

In contrast, imaging of all wells at a lower resolution (0.13 NA, 4 × ) with five z-stack images every 20 μm resulted in detection of a smaller number of aggregates with a larger variance leading to a lower Z′-factor (Fig. 4h, Supplementary Fig. 5). Even though the variance could be reduced by combining multiple wells, the Z′-factor of low-resolution data was still lower than what would be ideal and even lower than 1-well high-resolution data. Hence, it would be impossible, for example, to use high-speed plate readers with low NA and magnification for such screens.

### Assay validation and controls

To determine a good positive control and the best conditions for compound treatment of PolyQ35 animals, we tested the efficiency of several compounds studied in the literature. Specifically, we tested molecules that bind to various domains of Hsp90, and are proteasome inhibitors; 17-AAG, geldanamycin (Geld), radicicol (Rad), celastrol (Cel), and MG132 that activates HSF-1 expression, one of the downstream molecules regulated by Hsp90 activity^18^,^40^,^41^,^42^.

**Figure 5:**
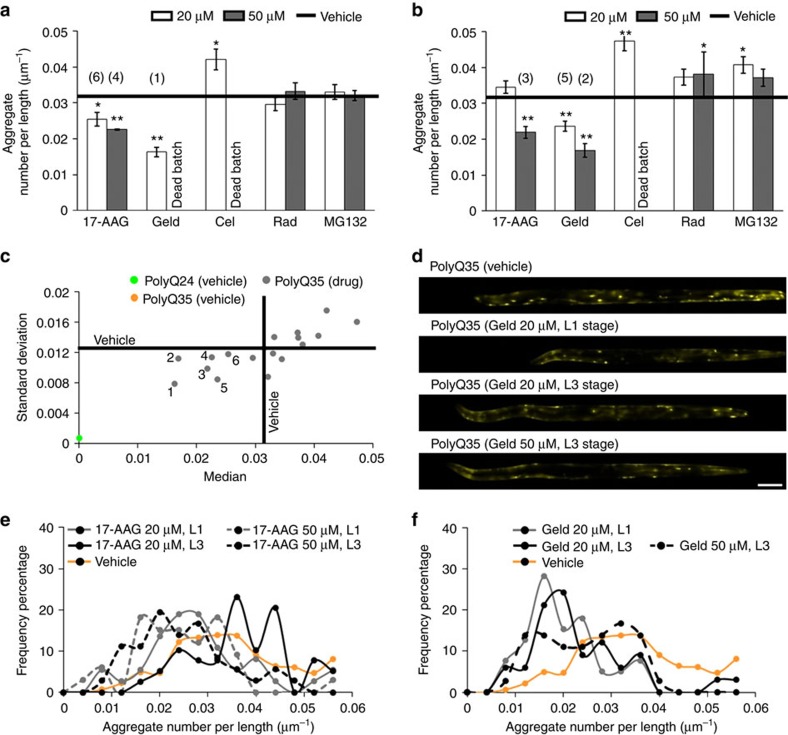
Assay validation with PolyQ aggregation model animals for drug screening. We tested a total of 20 different populations of PolyQ35 animals grown in 10 different batches and treated at 2 different stages with 5 different compounds: 17-AAG, Geldanamycin (Geld), Celastrol (Cel), Radicicol (Rad), and MG132. (**a**) Median aggregate numbers per unit length of the animals treated at L1 stage and imaged on D3 stage. (**b**) Median aggregate numbers per unit length of the animals treated at L3 stage and imaged on D3 stage. The black lines represent the median value for vehicle treated (0.5% DMSO) animals. (**c**) Standard deviation is plotted against median of aggregate numbers per unit length for each treatment condition. The positive results, identified as wells with lower median aggregate numbers per length and lower standard deviation values, are marked as: (1) (20 μM Geld, L1 stage), (2) (50 μM Geld, L3 stage), (3) (50 μM 17-AAG, L3 stage), (4) (50 μM 17-AAG, L1 stage), (5) (20 μM Geld, L3 stage), and (6) (20 μM 17-AAG, L1 stage). (**d**) Images of animals treated with different conditions and imaged inside the microfluidic chip. Scale bar is 100 μm. (**e**) Frequency percentage plot for aggregate numbers per unit length of PolyQ35 animals treated with vehicle and 17-AAG at L1 and L3 stages and with 20 and 50 μM concentrations. (**f**) Frequency percentage plot for aggregate numbers per unit length of PolyQ35 animals treated with vehicle and Geld at L1 and L3 stages and with 20 and 50 μM concentrations. Error bars in (**a**,**b**) are standard error of the mean (*n*≥33 animals). Statistical significance for individual compound was calculated within same stage with respect to the vehicle control using two-tailed *t*-test and represented as *P*<0.01 (*) and *P*<0.001 (**).

Among the five compounds, 17-AAG and Geld significantly reduced aggregate numbers per unit length in PolyQ35 animals in a dose-dependent manner (Fig. 5a,b). The results with 17-AAG were in agreement with previously published work that treated the same animal model with 50 μM of compound^18^. The other three compounds (Rad, Cel, and MG132) did not reduce aggregate phenotypes in our assay while they were found to be effective in *in vitro* cell culture models^18^,^42^,^43^,^44^. Treatment with 17-AAG and Geld during L1 stage was more effective than treatment during L3 stage; however, toxicity of Geld increased under these conditions (Fig. 5a,b). In agreement with published *in vitro* studies, 50 μM Cel treatment during L1 and L3 stages was also toxic^45^.

Although the median value of aggregates per unit length were lowered by 17-AAG and Geld, the percent of reduction was randomly distributed between individual animals in the population (Fig. 5c,d). PolyQ35 animals treated with higher concentration of 17-AAG and Geld compounds tended to have lower median values but a wider distribution for the aggregate number per unit length (Fig. 5e,f). Such variability was reported in populations treated with 50 μM 17-AAG (ref. 18). Aggregate parameters for animals treated with 50 μM Geld had a bimodal distribution, which could be due to inconsistent efficacy of Geld across the population resulting in a more heterogeneous population of mixed healthy and sick animals.

Since Geld was found to be the most efficacious of all five tested drugs, it was considered as the positive control for our large-scale drug screening. Furthermore, we selected to treat PolyQ35 animals at L3 stage with compound concentrations of 20 μM, since this dose of tested control drugs had good solubility and 100% survival rates. Positive hits were defined as compounds that reduced both median and standard deviation of aggregate numbers per unit length.

### Screening of FDA-approved compounds using our HTS platform

**Figure 6:**
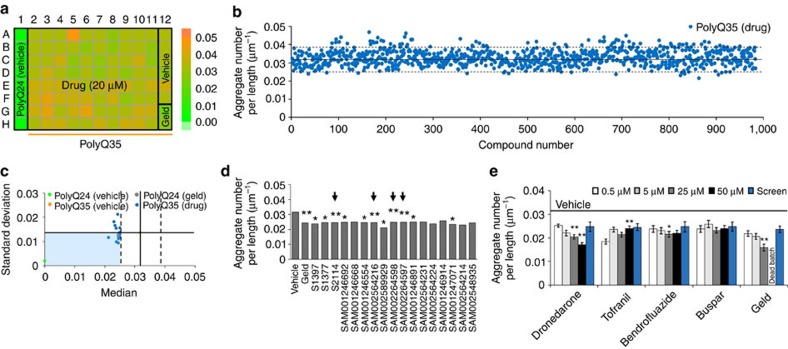
High-throughput screening of FDA-approved compounds. (**a**) An example heat map of median aggregate numbers per unit animal length and the loading map. (**b**) Summary plot of all 983 FDA-approved drug compounds as scored from 14 independent whole-chip experiments. The solid and dotted lines represent, respectively, the median and ±3 × standard deviation of the aggregate numbers per unit length of PolyQ35 animals treated with vehicle. (**c**) Summary of results from 17 drug compounds with reduced aggregate numbers having median values lower than the 3 × standard deviations of the vehicle control. (**d**) Statistical significance of these 17 drug compounds as calculated with respect to the vehicle control using two-tailed *t*-test (*n*≥33 animals except *n*=20, 25, and 15 for S1397, S1377, and SAM002589929, respectively). Four of the compounds (S2114, SAM002564216, SAM002264598, and SAM002264597) exhibited statistically significant lower median aggregate numbers per unit length (*P*<0.005) and are indicated by the arrows. (**e**) Results of hit validation experiments for these four compounds for animals treated at L1 stage at four different concentrations (0.5, 5, 25, and 50 μM). The blue bars represent the values obtained from the original screen as reference points. Error bars are standard error of the mean. Statistical significance for all different doses for individual compounds was calculated with respect to the lowest concentration using two-tailed, *t*-test (*n*≥70 animals except *n*=37 for Geld 25 μM). The statistical significance values are represented as *P*<0.05 (*) and *P*<0.005 (**).

To demonstrate the platform capability for large-scale drug screening, we performed HTS with 983 FDA-approved compounds from three different diverse libraries using 14 runs of full 96-well chips. Each run included up to 80 different compounds, 8 wells of vehicle-treated PolyQ24 animals, 6 wells of vehicle-treated PolyQ35 animals, and 2 wells of Geld-treated positive controls (Fig. 6a). Compounds were applied at L3 stage in LC and at 20 μM concentration. Animals were imaged at D3 stage and analysed using our automated image processing algorithm to estimate the aggregate parameters for each animal and extract an average value for each well.

The median aggregate numbers per unit animal length for all the 983 compounds are represented in a single plot (Fig. 6b). Each drug-treated population was normalized using vehicle controls as described in assay validation experiments for positive controls. From this initial analysis, we found 17 compounds having median aggregate numbers that were lower than the 3 × standard deviation value of the vehicle (Fig. 6c). Further analysis of statistical significance of these 17 compounds showed that only four (S2114, SAM002564216, SAM002264598, and SAM002264597) significantly reduced aggregate numbers of animals when compared to the vehicle control population (*P*<0.005, student *t*-test) (Fig. 6d and Supplementary Table 1). Seven other compounds resulted in a moderate reduction (0.005<*P*<0.05, student *t*-test). Although compound SAM002589929 had a lower median value, it had *P*=0.022 (student *t*-test) due to lower number of animals (*n*=15).

### Confirmation of positive hits

To validate the four hit compounds with the highest levels of statistical significance, we tested their dose-dependent response at four different concentrations (0.5, 5, 25, and 50 μM). We applied the chemicals at L1 stage instead of L3 to test if these compounds could work with a better efficacy. The results were in agreement with our observations from the screening assay performed in L3 stage, confirming that these were indeed positive hits (Fig. 6e). Only dronedarone showed a strong dose-dependent reduction in the number of aggregates per unit length of the animals and resulted in a 46% reduction at 50 μM as compared to vehicle-treated animals. Interestingly, L1 animals treated with 50 μM dronedarone could develop better as compared to the dead or unhealthy batches of animals when treated with 50 μM of our positive control, Geld (Supplementary Fig. 7). In summary, we confirmed four hits resulting in a hit rate of 0.4% from screening a library of 983 compounds. This rate of confirmed hits is comparable to the hit rate of a recent cell-based screen with ∼900,000 small molecules that resulted in 796 primary hits (0.09%) and 263 confirmed hits (0.03%)^18^.

## Discussion

In this work, we demonstrated for the first time a large-scale, robust microfluidic technology to image multiple populations of adult *C. elegans* at high-speed, while providing ∼1 μm imaging resolution. The automated platform enabled imaging the whole body of ∼4,000 animals in 15 different z-planes in 16 min. The device operates with a novel gasket system that immobilizes all 96 populations of animals in individual wells in less than 4 min using a single pressure input. Analogous flow rates in our optimized channel geometry and smart chip layout achieved uniform immobilization efficiency and invariable trapping location within each channel across the whole chip. The invariable trapping location enabled minimum and repeatable pre-calculated stage motions to capture images from a total of 384 different locations on the chip, four locations for each well, in a short amount of time. This aspect was crucial in minimizing errors in image acquisition and assisting in data extraction during the automated image processing.

The parallel channel design with optimized separation between channels allowed a higher data density per pixel. When imaging 15 stacks of about 4,000 animals at high-resolution, the amount of data can rapidly reach TBs if only a couple of worms were captured per image. On the other hand, our optimized system can collect 5,760 images of 8 MBs, resulting in 46 GB of data for each chip run.

Another important aspect of our chip design was the accommodation of *C. elegans* populations with developmental and phenotypical changes during drug treatment. Animals with altered phenotypes (for example, dumpy, long, and variable brood size) were trapped in different locations within the channel length. The wells of populations with drug treatments that arrested or killed all animals were marked in the associated log file, and the corresponding wells in the chip were filled with backup PolyQ35 animals. Filling these wells eliminated the pressure-driven flow imbalance and helped maintaining a constant high-pressure gradient across all other wells during imaging sessions.

It was necessary to develop a modified LC protocol to enable growth and treatment of large numbers of adult *C. elegans* for high-throughput studies. It is inefficient and difficult to grow *C. elegans* in large quantities on NGM plates because of (1) non-uniform chemical exposure due to precipitation in agar, (2) altered food concentration with time, (3) time-varying drug exposure and absorption on plate, and (4) time-consuming, multiple manual transfers of animals during late stage monitoring. Immobilization inside microfluidic chips of adult animals further requires the animals to be loaded in a clean liquid environment without any particulates to avoid clogging of small channels. In the new protocol, animals were grown and treated with different chemical compounds, and filtered in parallel to operate a debris-free condition in our HTS platform.

Using our new platform, we screened 983 FDA-approved clinical compounds using the PolyQ protein aggregation disease model for its relevance to the Huntington's disease in humans. Four of these compounds were found to reduce the aggregation parameters statistically below the vehicle-treated controls (*P*<0.005). One of these four compounds, dronedarone (S2114), is used in therapy for the treatment of patients with paroxysmal and persistent arterial fibrillation or atrial flutter^46^. The second compound, tofranil (SAM002564216), a strong serotonin reuptake blocker, is mainly used in the treatment of depression^47^. The third compound, bendrofluazide (SAM002264598), is used for treatment of hypertension. The fourth compound, buspar (SAM002264597), is an anxiolytic psychotropic drug to treat anxiety. All four hits were confirmed in validation screens using duplicate runs and tested for their dose response in batches treated at L1 stage. Among these four compounds, dronedarone showed a pronounced dose dependence reduction in aggregate numbers per unit body length when treated at L1 stage. Animals treated with dronedarone at 50 μM were also healthier and had lower degeneration as compared to the 50 μM Geld, and hence proved to show minimal toxicity response to high dose-treatment. Dronedarone can prove to be a better positive control for future screens as compared to the Geld. In the future, this assay can be used to study the mode-of-action of these positive hits and run a larger number of diverse drug libraries on this *C. elegans* model.

During the development of this platform and assay, we tested more than 2,500 different populations with 100,000 individual animals. We were able to screen the library of 1,000 compounds over a period of 3 months. During this period, we spent the majority of time for growing the synchronized populations of animals and carefully treating them with different compounds through tedious manual steps. Automated liquid handling systems can accelerate this process by handling multiple batches in parallel. We do not envision any difficulty in implementing such robotic systems in the flow process of our HTS platform.

The large-scale nature of our chip substantially reduced required fabrication time, resulting in orders of magnitude faster imaging and analysis than currently available systems. Current state-of-the-art serial microfluidic technologies would require enormous amount of fabrication and operational time to run 2,500 different chips to screen through all the individual populations. Even if the chips could be reused a few times after a thorough cleaning step, such a screening strategy would demand an enormous amount of fabrication and operation time and impossible to meet HTS requirements. Another major time-consuming step that we overcame was the image analysis. Using our software, we successfully reduced the data processing time by 1,000–10,000 × compared to the manual image analysis.

In summary, our platform provides a unique way to screen for subtle phenotypic changes in various *C. elegans* disease models at a cellular or sub-cellular level and at high speeds. With small modifications, the 96-well chip presented here can be adapted for use in plate readers and for testing different ages of animals at higher throughputs by incorporating a 384-well format. The ability to screen whole animals at the speed and cost of the *in vitro* cell-based assays, while providing an early indication on toxicity and absorptivity is the key for future cost-effective drug developments.

## Methods

### Chip fabrication

Microfluidic chips were fabricated using three-layer photolithography and single-layer soft-lithography techniques (Supplementary Fig. 1). The three-layer mould was fabricated on a 6-inch silicon wafer. The SU8 mould was treated with tridecafluoro-1,1,2,2-tetrahydrooctyl-1-trichlorosilane vapour (United Chemical Technologies) in a vacuum chamber at 40 °C to reduce surface adhesion during the soft-lithography process. To mould the microfluidic chip, PDMS (Dow Corning) and the curing agent were mixed in the ratio 10:1 and poured on the silanized SU8 mould. The wells were fabricated by positioning a 96-well PCR plate on top of the SU8 features^48^. The PDMS chip was cured at 70 °C for 2 h, peeled from the SU8 mould, and released from the PCR plate. The PDMS block was punched for exits (Syneo) and bonded to a 3/16 inch borosilicate glass substrate using 100 W oxygen plasma. The whole chip was finally cured at 70 °C for 6 h for complete sealing.

### Flow rate characterization

Microfluidic chips were fabricated with narrow input punches and coupled with metal couplers for flow rate measurements. A high-pressure buffer line was connected to one of the inlet punches while all other inlet punches were blocked using metal plugs to prime the chip. Flow rates were measured by collecting buffer through the exit for 20 s at different pressure values (2.5, 5.0, 7.5, and 10.0 psig). Output volume collection was repeated four times for every well and pressure value.

### Gasket and pressure system

The gasket system was comprised of two pieces: an aluminum (Gasket-1) piece and an acrylic (Gasket-2) piece (Supplementary Fig. 1f). The dimensions of Gasket-1 were designed to match the standard well plate dimensions and thus could fit in a flat-top motorized stage. Gasket-2 was machined with one buffer entry port and one air vent. Gasket-2 holds a thin layer of buffer layer on top of all 96 wells during chip operation. Two narrow lines were milled along the edge of larger sides of the rectangle to connect all six exit punches on each long side of the microfluidic chip. The top and the bottom gasket pieces were held tight using six screws to avoid buffer leakage during high-pressure steps. The chip was operated using filtered M9 from a 500 ml reservoir that was pressurized using compressed air controlled via one solenoid valve.

### Animal loading and immobilization in 96-well chip

All 96 wells were filled with ∼80 D3 adult worms in M9 buffer using an 8-channel pipette. The chip was loaded and clamped within the gasket system. After installation, the top gasket was filled slowly with M9 buffer. To initiate immobilization, we opened both exits (Exit1 and Exit2) simultaneously while initiating a computer controlled on/off pressure cycle. The whole 96-well chip with 3,840 traps was filled with animals in less than 4 min. Once the immobilization cycle was complete, the stage was moved to well A01 to initiate imaging algorithm.

### Automated image acquisition and hardware control

We developed an automation software using LabVIEW to control the CCD camera, the flat-top translational stage, and the 500 μm travelling range (±2 nm) piezo stage (MS2000, Applied Scientific Instrumentation) for automated imaging of all trapped animals (Supplementary Fig. 4a). The imaging was performed using an Olympus IX73 microscope that was equipped with a large area 15 × 15 mm^2^, 4 megapixel, 7.4 × 7.4 μm^2^ pixel size, 15 frames per second maximum frame rate CCD camera (MegaPlus ES4020, Princeton Instruments). Bright field and fluorescence images were acquired using 2 × (0.06 NA) and 10 × (0.3 NA) objectives, respectively. The software controlled the exposure time of the camera and its synchronization with the movement of a motorized stage platform. The exposure time of the camera was optimized to achieve the highest imaging speed to achieve images with good signal to noise level. The scan area was optimized to capture 10 channels in each FOV and within four translations per well for whole 96-well chip.

To correct for the misalignments, the algorithm calibrates XY offset (*θ*_XY_) and XZ tilt (*δ*_XZ_ and *δ*_YZ_) of the channel design with respect to the stage axes (Supplementary Fig. 4d–f). The bending of the glass substrate (*Δ*) under higher gasket pressure caused the trapped animals and their cells to move away from the imaging plane by up to 70 μm across the chip, making it impossible to score for the phenotypes (Supplementary Fig. 5a–d). Incorporating all three corrections (*θ*_XY_, *δ*_XZ_, *δ*_YZ_, and *Δ*(*x*,*y*)) automated imaging algorithm acquired 15 z-stack images from all 96 wells in less than 16 min generating ∼40 GB of images that were stored as 16-bit TIFF images. A total of 25 chips operated in this study generated approximately 1.25 TB of images that were analysed to obtain data from approximately 100,000 D3 adult *C. elegans*. The data was saved in two different computers in Ben-Yakar Lab computers as well as the University's centralized storage units at the Texas Advanced Computer Center for long-term storage.

### Automated image analysis

We developed an automated image analysis program based on image processing algorithms (Fig. 4a–e, Supplementary Fig. 6). In addition a GUI program was developed to allow the user to rapidly load and process saved image stacks at different locations per well from a specific screening experiment with minimal user input at the beginning. All image stacks from a particular well and four different locations were loaded, merged, and summed up to increase the contrast between the cells and surrounding areas. The filtered image was projected to an axis perpendicular to the length of the worm and searched for peaks to isolate single worms immobilized inside the microfluidic channel. All the planes through all the image stacks for each animal were analysed to identify the best focal plane with maximal variance after applying a Laplacian Gaussian filter^49^,^50^. The whole image in the same focal plane was used to identify the length of the worm and all the aggregate present in the whole animal image using a particle filter. The worm length was found by projecting the cropped image on the axis parallel to the channel length and finding the region within the pixels with intensity higher than 2 × above the background. The intensity threshold was estimated from the vehicle-treated PolyQ35 animals so that 3% of bright pixels lie higher than the threshold value. Using this threshold value, animals in drug-treated wells were converted to binary images. Regions of higher gradient were found by implementing a Laplacian of Gaussian filter (*σ*=7) to the best-focused images and converting them to binary images. The two binary images were multiplied together so that only regions of high intensity and high gradient were chosen. Animals were scored for aggregate parameters such as number of aggregates, length of the animal, and size of each aggregate. The data of phenotypical scores were calculated as median aggregate number per unit length for each well and saved in a multi-dimensional array to be extracted for statistical analysis. The median values were plotted in a window to show the outcome of screens.

The algorithm followed the same structure for images collected under 4 × objective except few minor changes. First, because the FOV was different, the merged images were cropped to only include those worms that are within the traps. Second, the Laplacian of Gaussian filter was reduced in size (*σ*=7/2.5) to reflect the 2.5 × de-magnification from 10 × to 4 × .

### Statistical analysis

Raw images were analysed and the scores were compiled into arrays to be analysed for statistical tests. Aggregate parameters were analysed for statistical significance using two-tailed student *t*-test with unequal variance. Where measurement was repeated in multiple batches, mean values were calculated to represent in the graph. The quality of the screen was quantified by calculating the Z′-factor^51^.

### Chemicals and drug libraries

All chemicals for *C. elegans* maintenance were bought from Fisher Scientific and Sigma-Aldrich. Drug compounds 17-AAG, Geldanamycin (Geld), Celastrol (Cel), Radicicol (Rad), and MG132 were purchased from Enzo Life Sciences. 5-Fluoro-2-deoxyuridine (FUdR) and DMSO was purchased from Sigma-Aldrich. All the compounds were dissolved in 100% DMSO at 10 mM concentration and used at different dilutions in the worm culture as mentioned in the text. FUdR was dissolved in water.

We tested all five drug compounds (17-AAG, Geld, Cel, Rad, MG132) with 20 and 50 μM concentrations. To identify the best conditions for delivering the compounds, we tested animals treated in two different ages. In the first batch, all the animals were treated from L1 stage, while in the second batch they were treated from L3 stage (24 h after plating L1). To avoid new progenies in the culture, we added 200 μM FUdR at L4 stage of animals in both batches. Animals treated with only 0.5% DMSO were considered as a vehicle control for the aggregation parameters. Animals were filtered using our modified LC protocol, imaged in our HTS platform using the automated image acquisition, and analysed for aggregate parameters using the image processing algorithms. For each treatment condition, median aggregate values of animals treated with vehicle control were normalized to match the median value (0.0318) obtained from our assay characterization experiments. Each drug-treated population was then normalized with the same factor.

Three drug libraries with FDA-approved compounds were used for this study with total 983 compounds; custom selected bioactive compounds, in which their bioactivity and safety were confirmed by preclinical research and clinical trials (256 compounds; Selleck Chemicals), NIH clinical collection I (446 compounds; Evotec, South San Francisco, CA, Cat No NCC-104), and NIH clinical collection II (281 compounds; Evotec, South San Francisco, CA, Cat No NCC-201). All the libraries were prepared from 10 mM stock plates using a liquid handling system at UT Austin's TTDDDP (Targeted Therapeutic Drug Discovery and Development Program) facility. The dilution plates were prepared at 4 mM concentrations in 100% DMSO and in 384-well plates. The vehicle control wells were filled with 100% DMSO alone. The dilution plates were stored frozen at −80 °C. Dilution plate was thawed and 2.5 μl from each quadrant of the 384-well drug plate was used at a final concentration of 20 μM in the *C. elegans* culture after 24 h of plating. For hit validation tests, we procured all four compounds from commercial vendors: Dronedarone (Selleck), Tofranil (Sigma), Bendrofluazide (Sigma), and Buspar (Sigma). These compounds were dissolved in 100% DMSO to prepare 10, 5, 1, and 0.1 mM stock solutions. A volume of 2.5 μl of the stock solutions was used at a final concentration of 50, 25, 5, and 0.5 μM in the LCs.

### *C. elegans* strains and maintenance

*C. elegans* were grown and maintained on NGM agar plates with bacteria at 20 °C according to the standard method^52^. We used the following *C. elegans* strains in this work: PolyQ24 AM138 *rmIs*[*unc-54p::Q24::YFP*], PolyQ35 AM140 *rmIs132*[*unc-54p::Q35::YFP*], and PolyQ40 AM141 *rmIs132*[*unc-54p::Q40::YFP*]. PolyQ24 and PolyQ35 animals were labelled with Q24::YFP and Q35::YFP in the body wall muscle, respectively. PolyQ24 animals had soluble YFP signal all throughout their D3 age while PolyQ35 animals showed soluble YFP signal in early developmental stages and aggregated YFP puncta at day three (D3) adult stage.

### Large-scale liquid culture of *C. elegans* for HTS

Four healthy L4 hermaphrodites were transferred to a 10 cm diameter NGM plate seeded with HB101 bacteria and grown for 7 days at 20 °C. The gravid animals were bleached and eggs were incubated at 20 °C in a 360 degree rotor for 24 h for hatching. Age synchronized L1s were filtered and plated in four 24-well plates with approximately 120 animals per well. Animals were incubated with 500 μl of HB101 bacterial culture in S medium at 20 °C for 48 h. Fresh bacterial culture of 250 μl volume and 200 μM sterility drug (FUdR) was added to every well. Appropriate drug compounds were added in designated wells at known concentrations. The plates were incubated for 72 h until the worm grew to D3 adult stage. All 24-well plates were filtered and individual animal populations were loaded into different wells with a 50 μl volume on a primed chip for high-throughput imaging.

### Data availability

The data that support the findings of this study are available from the corresponding author on request.

## Additional information

**How to cite this article:** Mondal S. *et al*. Large-scale microfluidics providing high-resolution and high-throughput screening of *Caenorhabditis elegans* poly-glutamine aggregation model. *Nat. Commun.* **7,** 13023 doi: 10.1038/ncomms13023 (2016).

## Supplementary Material

Supplementary Information Supplementary Figures 1-7, Supplementary Table 1, Supplementary Reference

Supplementary Movie 1 Trapping movie with the *C. elegans* animals.
