# Supplementary material for: Large-scale microfluidics providing high-resolution and high-throughput screening of Caenorhabditis elegans poly-glutamine aggregation model
Source: Nat Commun. 2016 Oct 11;7:13023. doi: 10.1038/ncomms13023 (PMC5062571; doi:10.1038/ncomms13023)
Supplement: Supplementary Information — Supplementary Figures 1-7, Supplementary Table 1, Supplementary Reference [file ncomms13023-s1.pdf]

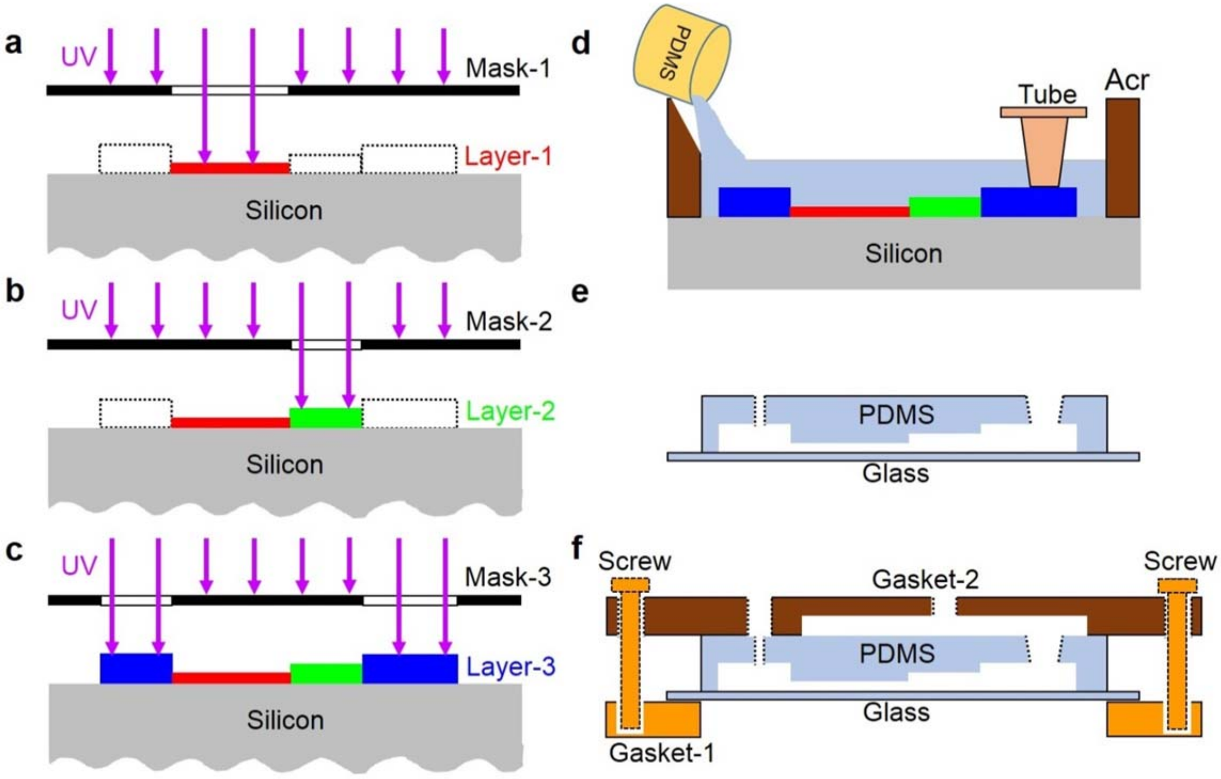

Supplementary Figure 1: Schematic of large-scale microfluidic chip fabrication and gasket assembly system. Multi-layer photo-lithography is used to fabricate Layer-1 (**a**), Layer-2 (**b**), and Layer-3 (**c**) on SU8 photo-resists using high-resolution photo-masks Mask-1, Mask-2, and Mask-3, respectively. Three layers are developed with three different heights to obtain desired aspect ratios on the SU8 mold. (**d**) PDMS mixture (10:1) is poured on the SU8 layer using an acrylic support (Acr) on edges and with silanized 96-well PCR tubes placed on top of the well pads to form well structures. (**e**) The PDMS mold is cured at high temperature, peeled off from the silicon substrate, punched holes for external connections, and bonded on to a glass substrate. (**f**) A bonded chip is mounted within a gasket assembly (Gasket-1 and Gasket-2) and clamped using screws. Holes in the top gasket helped to couple external connections for fluid inlets and outlets.

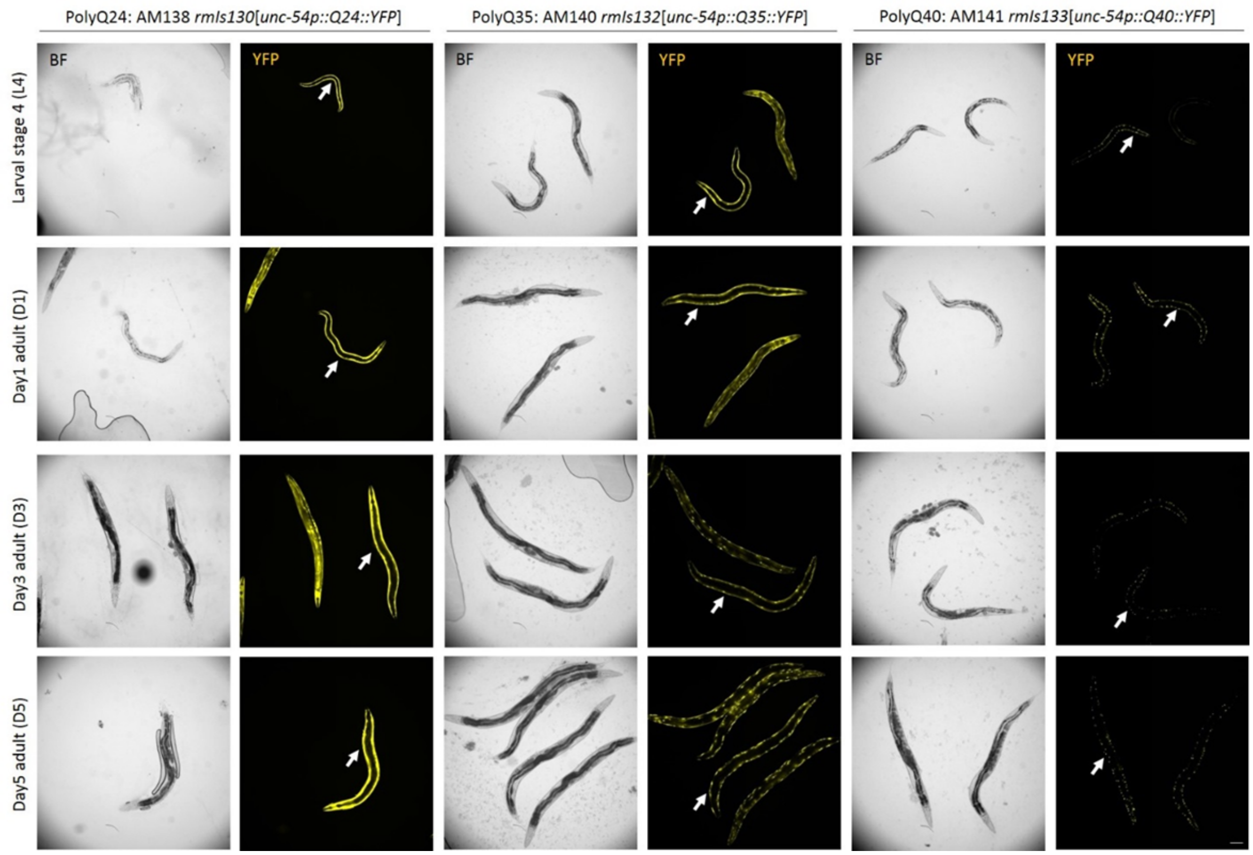

Supplementary Figure 2. Age dependent aggregate formation in body wall muscles found in different *C. elegans* strains growing in liquid culture. Three different strains PolyQ24, PolyQ35, and PolyQ40 were grown in liquid culture and imaged at different developmental stages. PolyQ24 strain was selected as healthy since it did not show age dependent aggregation. PolyQ40 animals had the aggregates very early on in their development. PolyQ35 was selected for this study since it showed a clear increase in aggregate number with development. Scale bar is 100 μm.

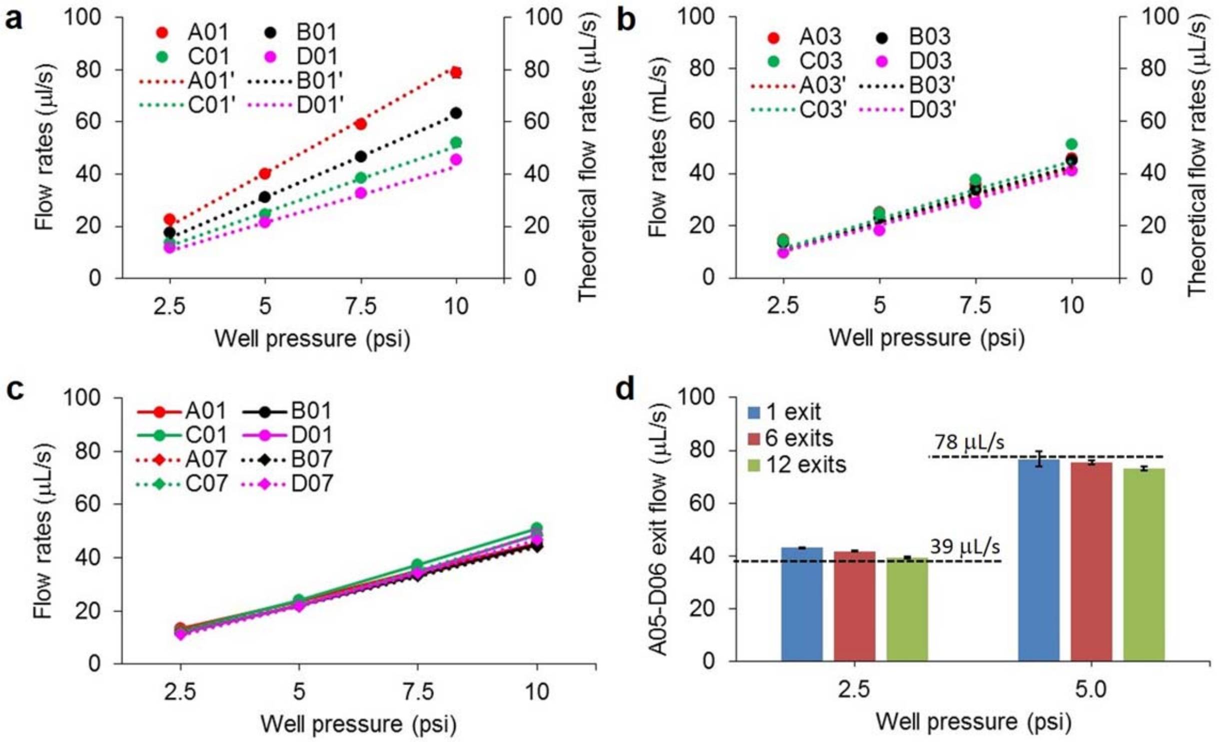

Supplementary Figure 3: Flow rate characterization of chips with 8- and 96-wells. (**a**) The flow rates for an 8-well chip with identical channel widths. (**b**) The flow rates of an 8-well chip with an optimized varying channel dimensions to achieve similar flow rates. Experimental data are represented by circles and estimated theoretical values are represented by dashed lines showing a good agreement. Hydraulic resistance of a channel section with length L and rectangular cross section $W \times H$ (width × height) were calculated using the equation[1] $R = \frac{12\mu L}{WH^3}[1 - \frac{H}{W}(\frac{192}{\pi^5}\sum_{n=1,3,5}^{\infty}\frac{1}{n^5}\tanh(\frac{n\pi W}{2H}))]^{-1}$. Assuming channels are filled with water of viscosity ($\mu = 8.94 \times 10^{-4}$ N-s/m^2), we calculated the equivalent resistances of all 40 parallel traps and the channels connecting them to the central exit (represented as R_A, R_B, R_C, and R_D in **Fig. 2g**). Resistances between the junctions along the central exits are calculated separately as R_{23}, R_{34}, R_{45}, and R_{50}. For a given applied pressure $P_{1,}$ we iteratively optimized hydraulic resistances to achieve similar values for the flow rates (Q_A, Q_B, Q_C, and Q_D) using an equivalent circuit (**Fig. 2h**). (**c**) The flow rates from wells at corners (A01-D01) and in the center (A07-D07) of the 96-well chip. Flow rates in (a-c) are measured at 2.5, 5.0, 7.5, and 10.0 psi well pressures. (**d**) Flow rates of channels connecting wells A05 – D06 with 2.5 and 5.0 psi gasket pressure, under single exit, 6 exits, and 12 exits open. Data represented as mean ± SD (n = 4).

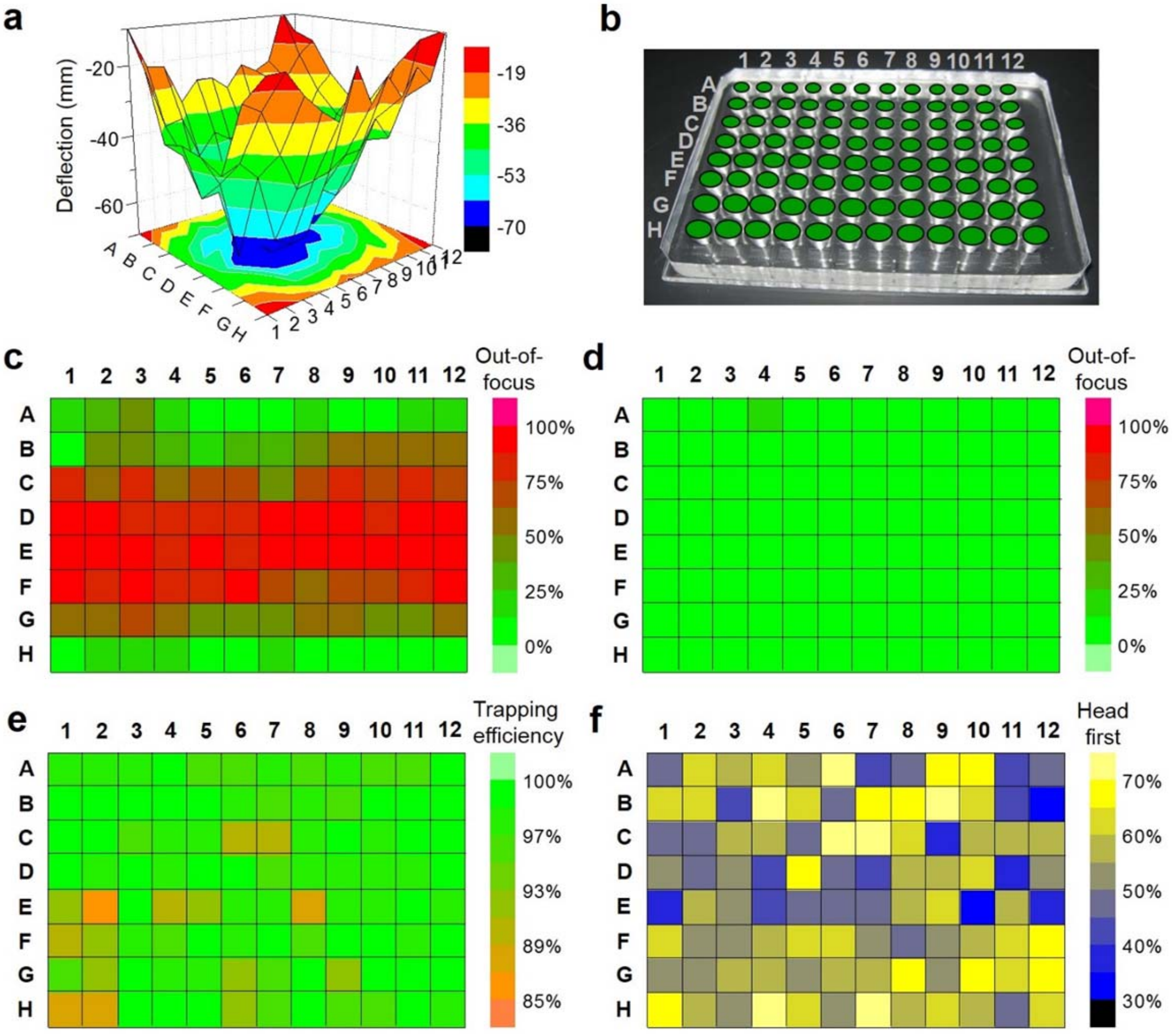

Supplementary Figure 4: Glass bending in 96-well chip under a constant gasket pressure. (**a**) A 3D color map of the amount of glass bending measured from a 96-well chip under 5.0 psi of gasket pressure. The color code on the side bar represents the height of the bending in micrometers. (**b**) Image of a 96-well chip with image analysis output represented by a circle for every well. (**c**) Plot representing the 96-wells showing the fraction of animals failed to appear in focus for cellular fluorescence signal without bending correction during imaging. The color code represents the percentage of out-of-focus images with the green indicating all animals being imaged in focus. (**d**) A large fraction of animals is in focus with correction algorithm for substrate bending for the same loading. (**e**) The immobilization efficiency map of an individual experiment shows that most wells' channels were filled with an efficiency greater than 93%, while a few wells (15%) were filled with efficiency between 85 – 93%. The filling efficiency was affected by a few random errors such as, errors in punching inlets on the chip, channels getting

clogged by eggs, random dust particles in a well, and bad channel shape due to delamination of SU8 mold. A strong clamping of the top gasket was also leading to poor efficiency at the edges of the chip, but we solved this problem by fine tuning the degree of clamping in subsequent experiments and a better understanding of the protocol. (**f**) The head-tail orientation of the trapped animals. The color bar indicates the percentage of animals immobilized with head first inside the immobilization channel.

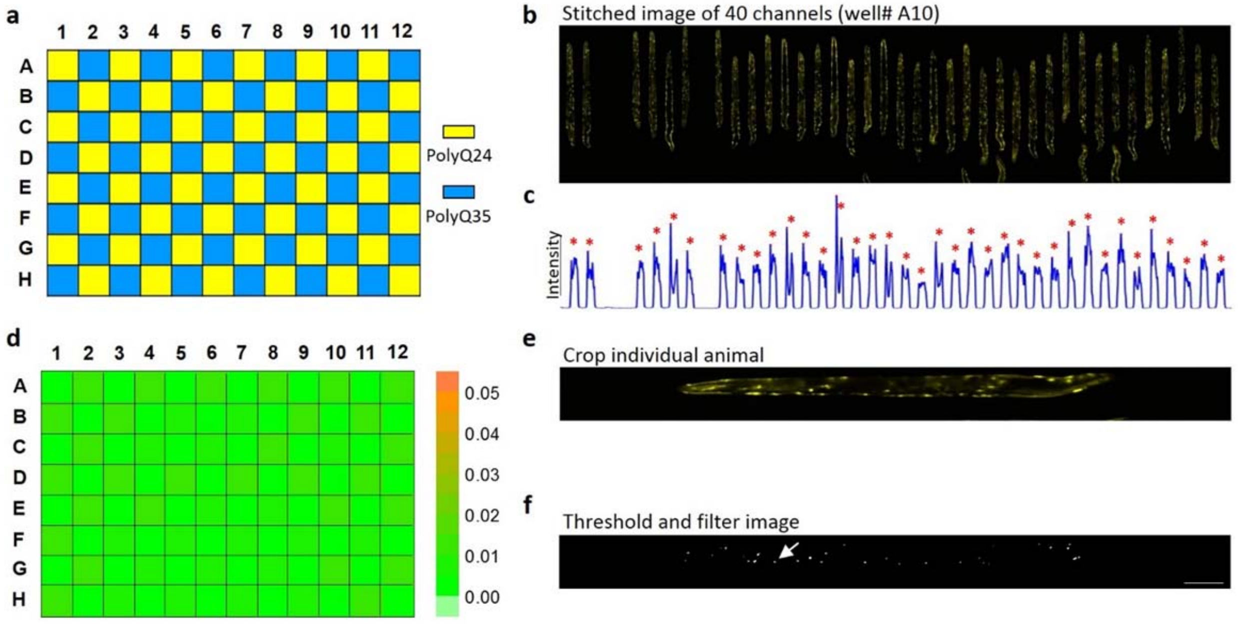

Supplementary Figure 5: Imaging at lower resolution identifies less number of aggregates with higher variation. (**a**) Loading map of PolyQ24 and PolyQ35 animals in a 96-well chip. (**b**) Stitched image of all 40 channels acquired with 0.13 NA, 4× objective. (**c**) One-dimension profile of the device to identify channels with trapped animals. (**d**) Average aggregate number per unit length map for the whole chip. (**e**) Cropped image of an individual channel with one single animal. (**f**) Threshold and filtered image of the same animal. Scale bar is 100 μm.

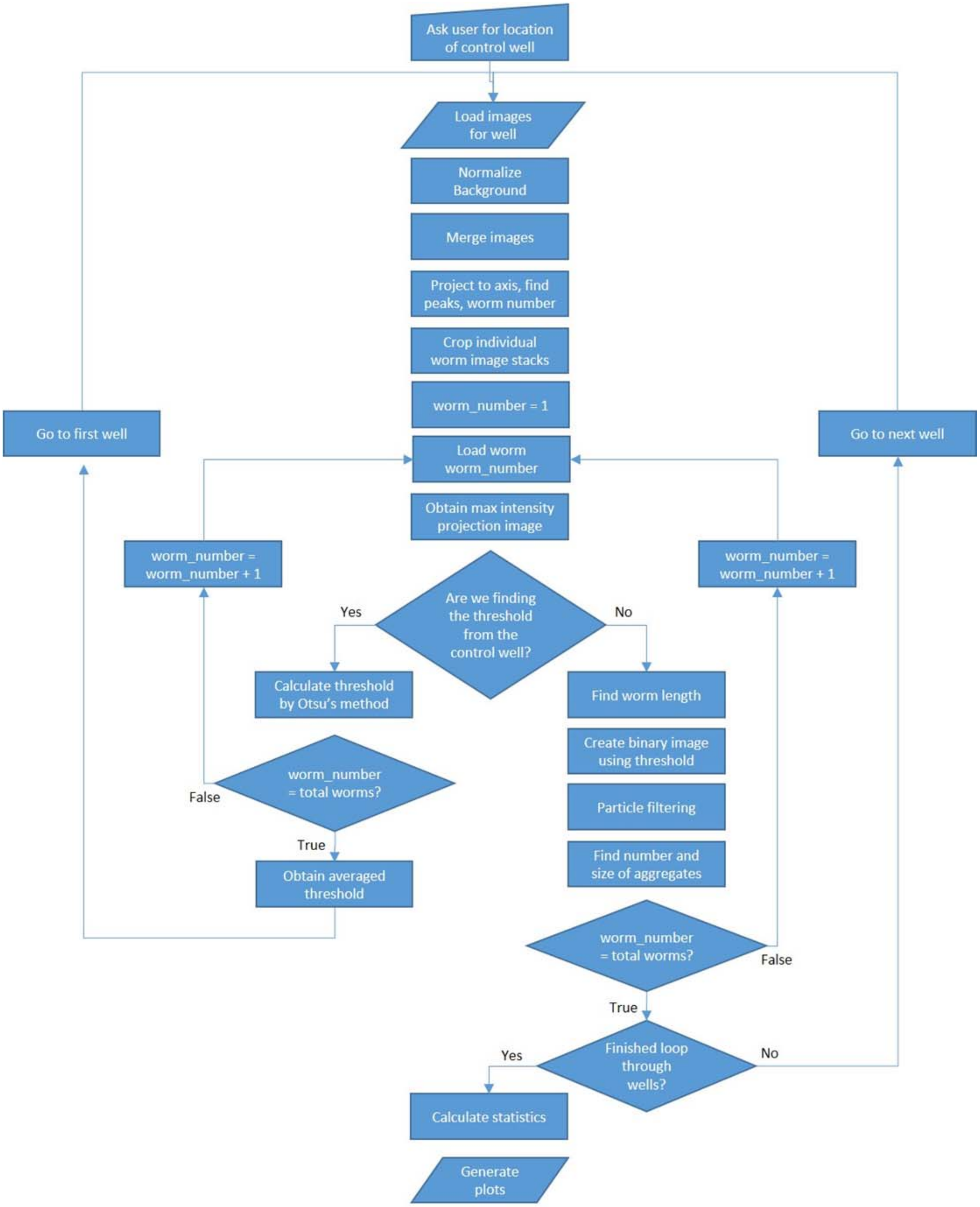

Supplementary Figure 6: Image processing flow chart. Flow chart for image processing algorithm to identify and quantify aggregate parameters in device immobilized *C. elegans*.

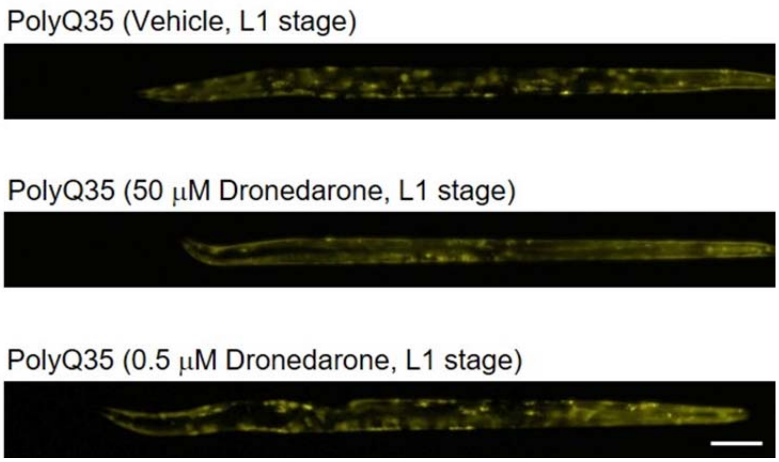

Supplementary Figure 7: Hit verification results. Images of *C. elegans* treated with 0.5% DMSO (vehicle), 0.5 μM dronedarone, and 50 μM dronedarone starting at L1 stages. Scale bar is 100 μm.

Supplementary Table 1: Summary of all 17 compounds having median aggregate numbers lower than the 3 × standard deviation value of the vehicle.

**Compound name**	**Median**	***p*-value[1]**
Geld	0.024356	<0.001 (**)
S1397	0.023847	0.006 (*)
S1377	0.024625	0.015 (*)
S2114	0.024688	0.002 (**)
SAM001246692	0.02469	0.040 (*)
SAM001246668	0.024964	0.092
SAM001246554	0.024534	0.006 (*)
SAM002564216	0.024507	<0.001 (**)
SAM002589929	0.02122	0.022 (*)
SAM002264598	0.024692	0.003 (**)
SAM002264597	0.024719	0.001 (**)
SAM001246891	0.024927	0.032 (*)
SAM002564231	0.024896	0.054
SAM002564224	0.023799	0.113
SAM001246914	0.025734	0.485
SAM001247071	0.023299	0.029 (*)
SAM002564214	0.02301	0.126
SAM002548935	0.024315	0.317

[1]Statistical significance for all different compounds was calculated with respect to the vehicle control using two-tailed t-test ($n \geq 33$ animals except $n = 20$, 25, and 15 for S1397, S1377, and SAM002589929). The statistical significance values are represented as $p < 0.05$ (*) and $p < 0.005$ (**).

Supplementary Reference:

1. Kleinstreuer, C. Microfluidics and Nanofluidics: Theory and Selected Applications. (Wiley, 2013).
